# Supplementary figures and images for: In silico repurposing of a Novobiocin derivative for activity against latency associated Mycobacterium tuberculosis drug target nicotinate-nucleotide adenylyl transferase (Rv2421c)
Source: PLoS One. 2021 Nov 2;16(11):e0259348. doi: 10.1371/journal.pone.0259348 (PMC8562812; doi:10.1371/journal.pone.0259348)

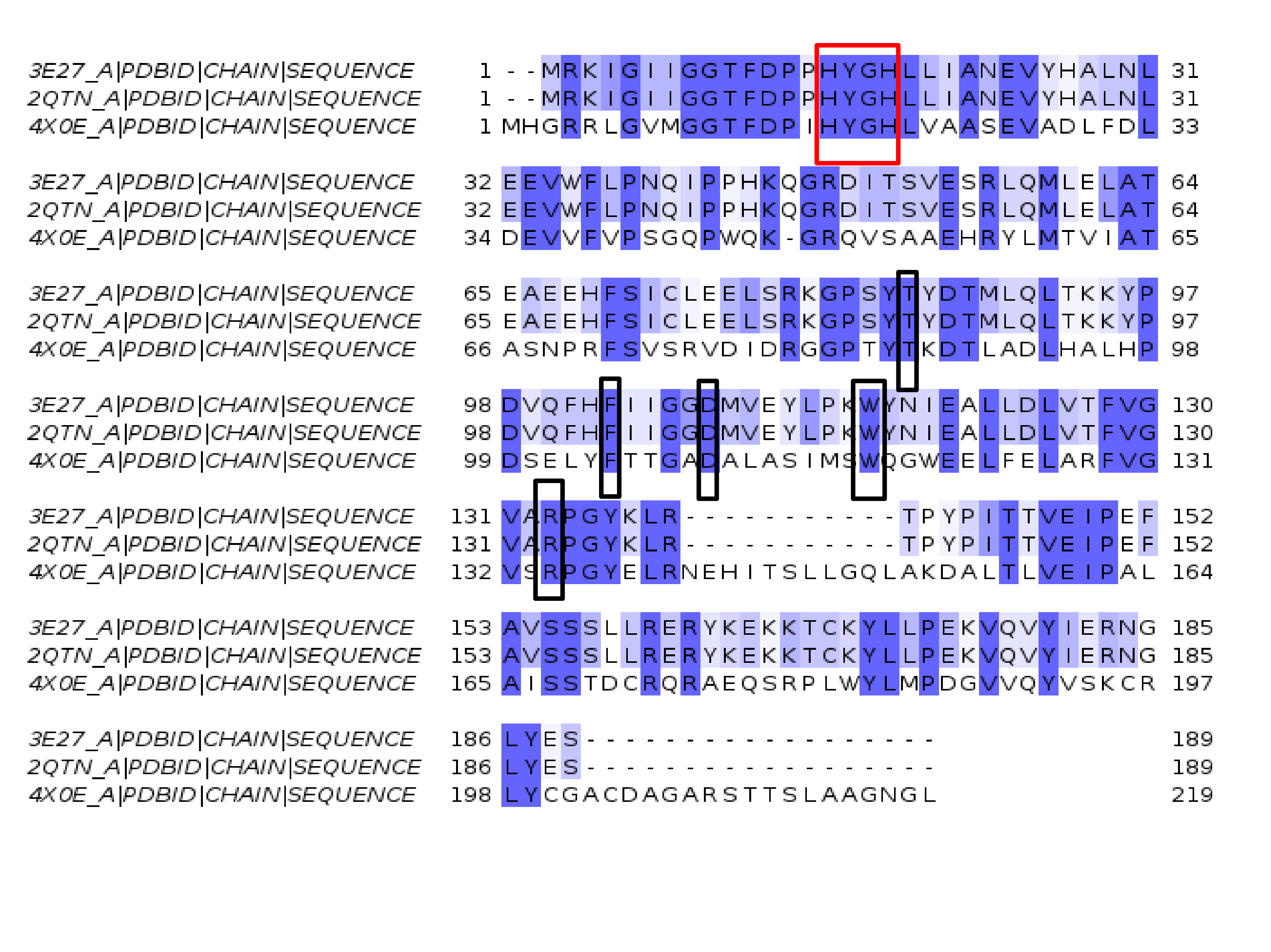

Supplement: S1 Fig — PDBID: 2QTR crystal Structure of nicotinate mono-nucleotide adenylyl transferase from B. anthracis, PDBID: 3E27 Nicotinic acid mononucleotide (NaMN) adenylyl transferase from B. anthracis: product complex. The alignment was performed using CLUSTALW v2.1 and was visually represented using Jalview v2.3. Shown in the red box are highly conserved signature sequence motif [(H/T)XGH]. DND active site residues are shown in black boxes. (TIFF) [file pone.0259348.s001.tiff]

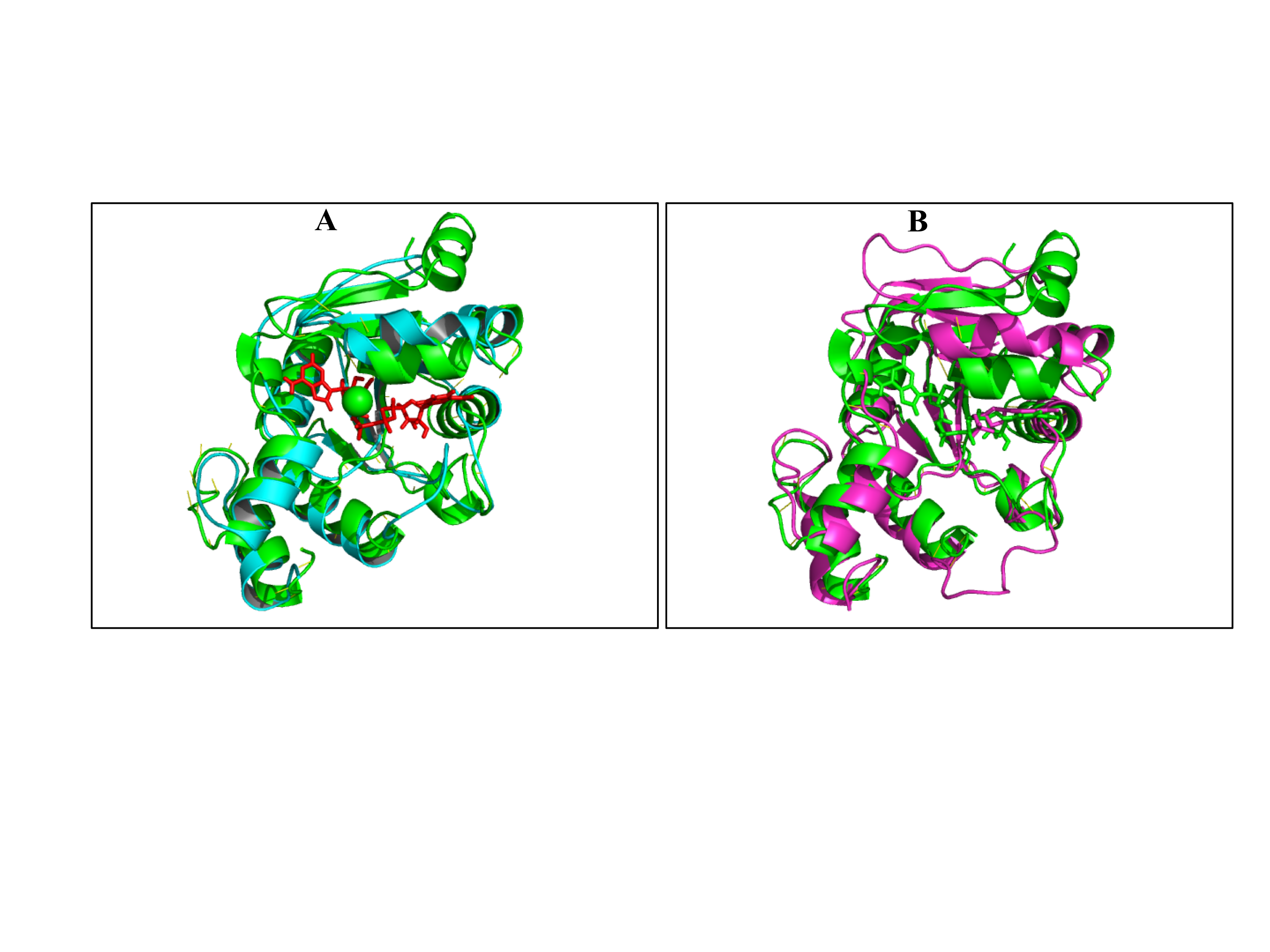

Supplement: S2 Fig — A) The green structure represents Rv2421c and cyan is 3E27 solved in complex with nicotinic acid adenine dinucleotide (DND). Substrate DND (red) shown as a stick representation and MG (green) shown as a sphere RMSD = 1.261Å.B) The green structure represents Rv2421c and in pink is 2QTN solved in complex with nicotinate mononucleotide (NCN). Substrates DND (green) and NCN (cyan) shown as stick representations. RMSD = 1.502Å. (TIFF) [file pone.0259348.s002.tiff]

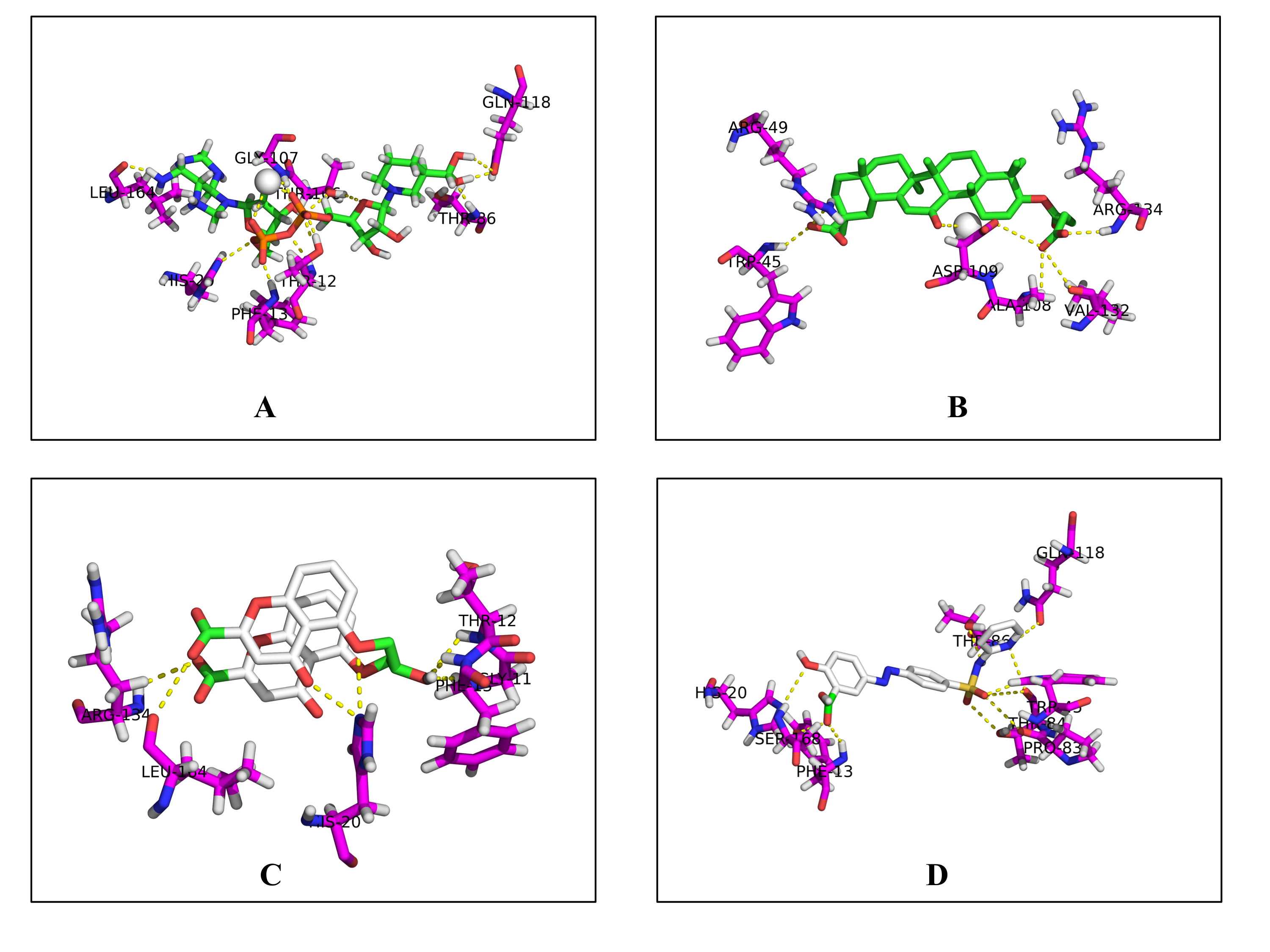

Supplement: S3 Fig — A) substrate DND is displaying eight hydrogen bond interactions, one hydrophobic contact and one MG ionic interaction with Rv2421c residues. B) Carbenoxolone disodium salt is displaying two hydrogen bond interactions and three hydrophobic contacts with Rv2421c residues. C) Cromolyn disodium salt is displaying three hydrogen bond interactions and two hydrophobic contacts with Rv2421c residues. D) Sulfasalazine is displaying four hydrogen bond interactions, three hydrophobic contacts and one pi-pi stacking interaction with Rv2421c active site residues. The dashed yellow lines represent hydrogen bonds. (TIFF) [file pone.0259348.s003.tiff]

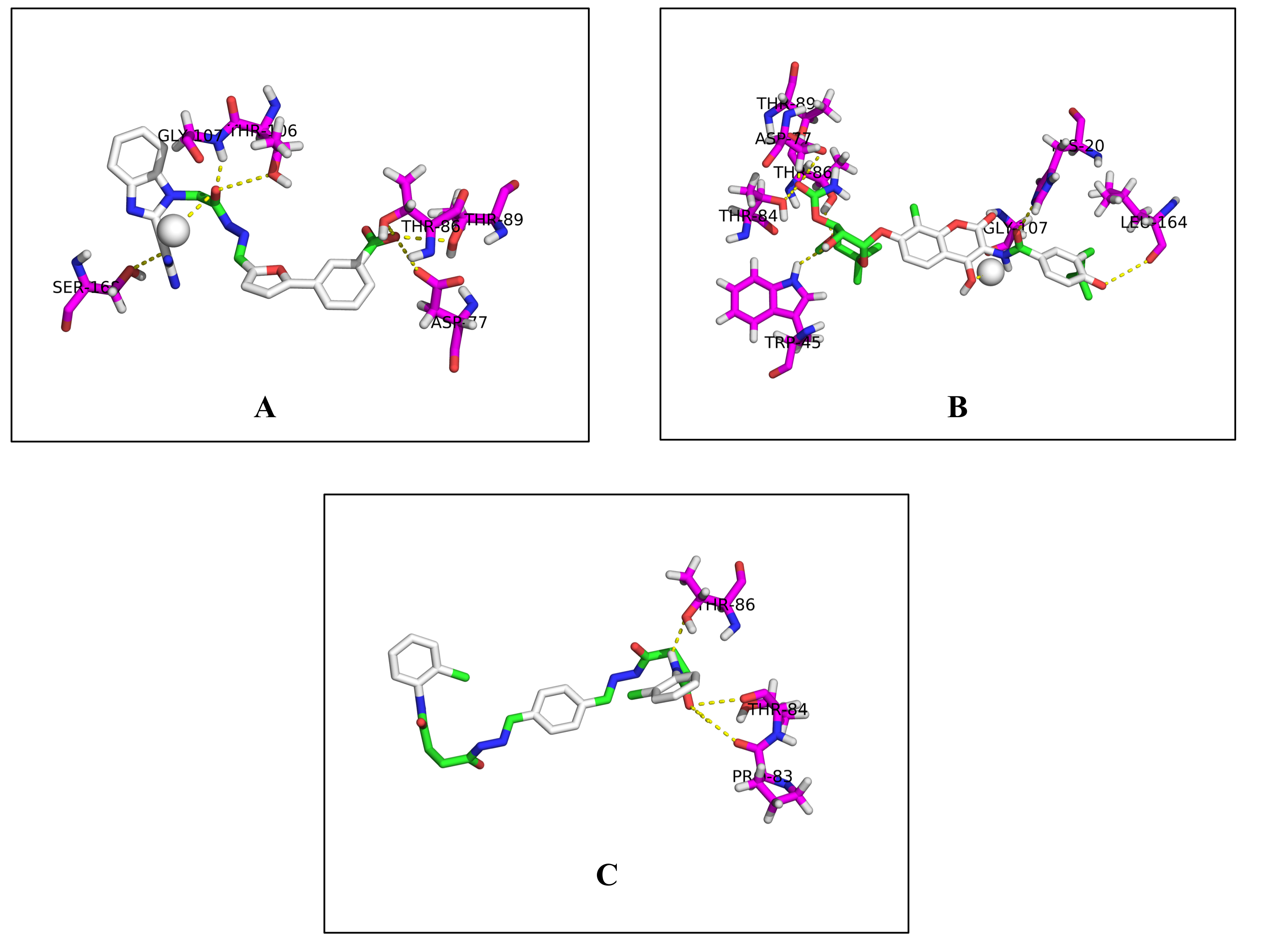

Supplement: S4 Fig — A) novel ZINC13544129 is displaying five hydrogen bond interactions and three hydrophobic contacts with Rv2421c residues. B) Novobiocin disodium salt is displaying four hydrogen bond interactions, two hydrophobic contacts and one MG ionic interaction with Rv2421c residues. C) The known NadD E.coli inhibitor ZINC58655383 is displaying three hydrophobic contacts and one pi-pi stacking interaction with the binding site residues of Rv2421c. The dashed yellow lines represent hydrogen bonds. (TIFF) [file pone.0259348.s004.tiff]

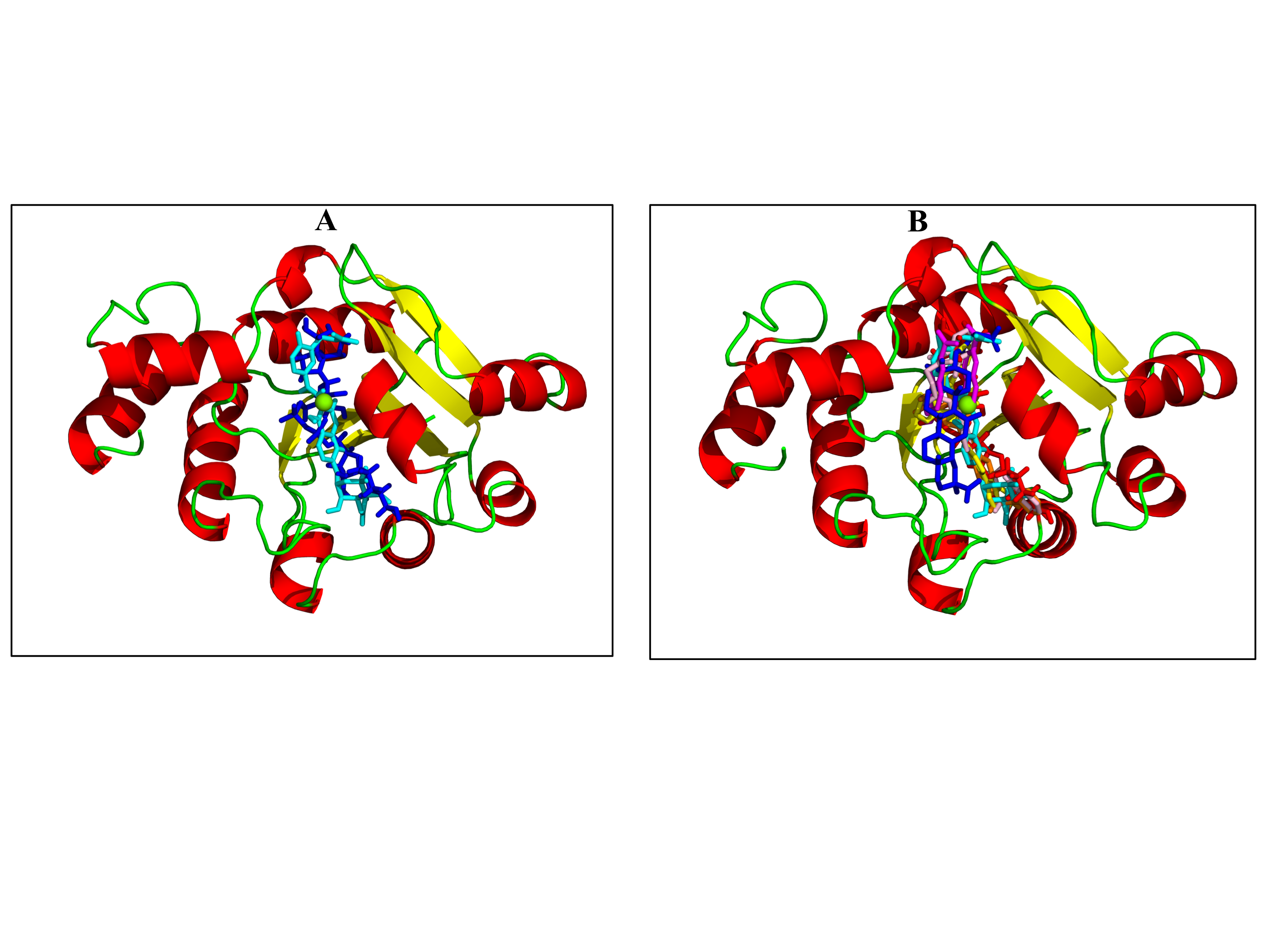

Supplement: S5 Fig — A) Similar binding pose for DND (blue stick) vs Novobiocin sodium salt (cyan stick). B) Different binding poses for Carbenoxolone disodium salt, Cromolyn disodium salt, Sulfasalazine, ZINC13544129, Novobiocin salt vs DND. Compound colouring schemes shown as sticks: DND is red, MG ion is green sphere, Carbenoxolone disodium salt is blue, ZINC13544129 is yellow, Cromolyn disodium salt is magenta, Novobiocin sodium salt is cyan, Sulfasalazine is orange and ZINC58655383 is in light pink. (TIFF) [file pone.0259348.s005.tiff]

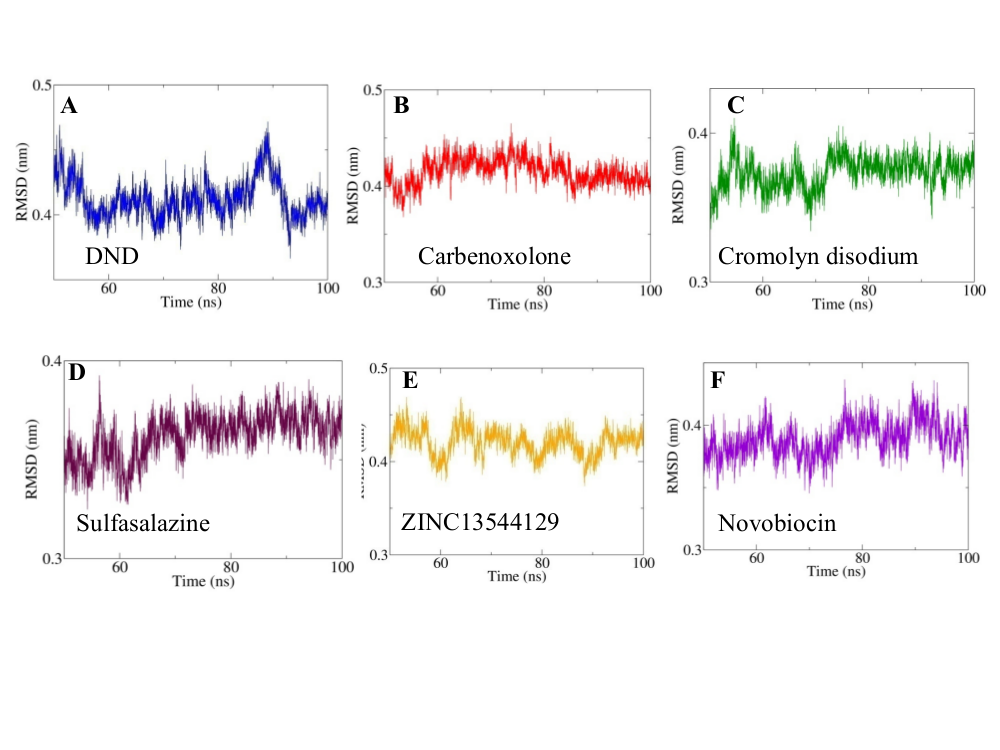

Supplement: S6 Fig — A) DND, B) Carbenoxolone disodium salt, C) Cromolyn disodium salt, D) Sulfasalazine, E) ZINC13544129 and F) Novobiocin salt. (TIFF) [file pone.0259348.s006.tiff]

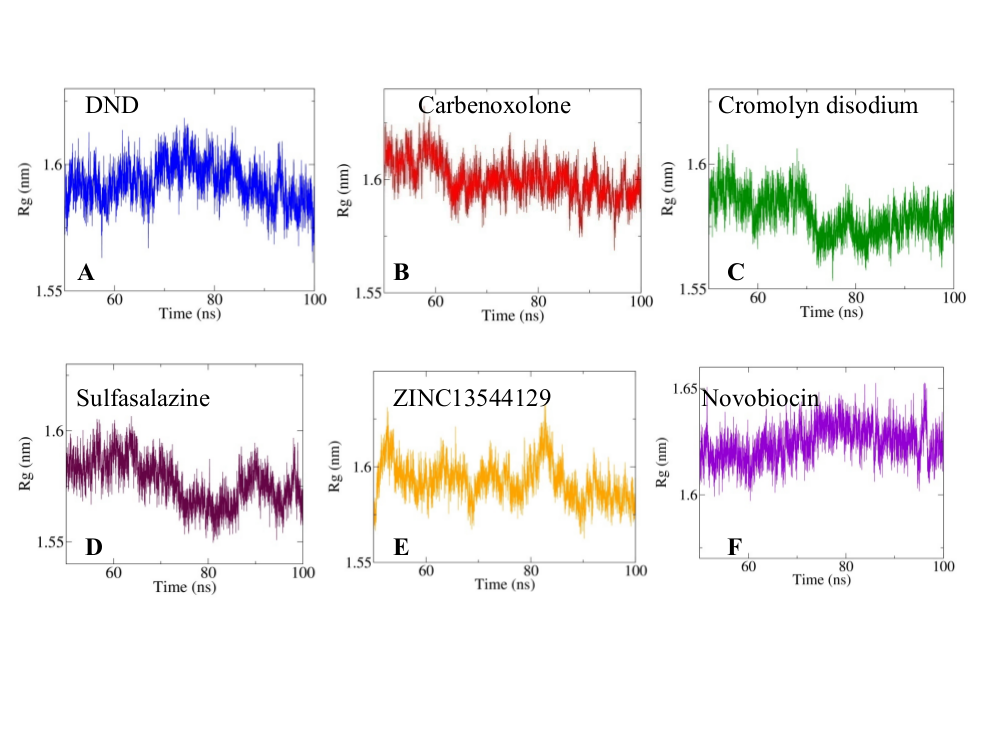

Supplement: S7 Fig — A) DND, B) Carbenoxolone disodium salt, C) Cromolyn disodium salt, D) Sulfasalazine, E) ZINC13544129 and F) Novobiocin salt. (TIFF) [file pone.0259348.s007.tiff]

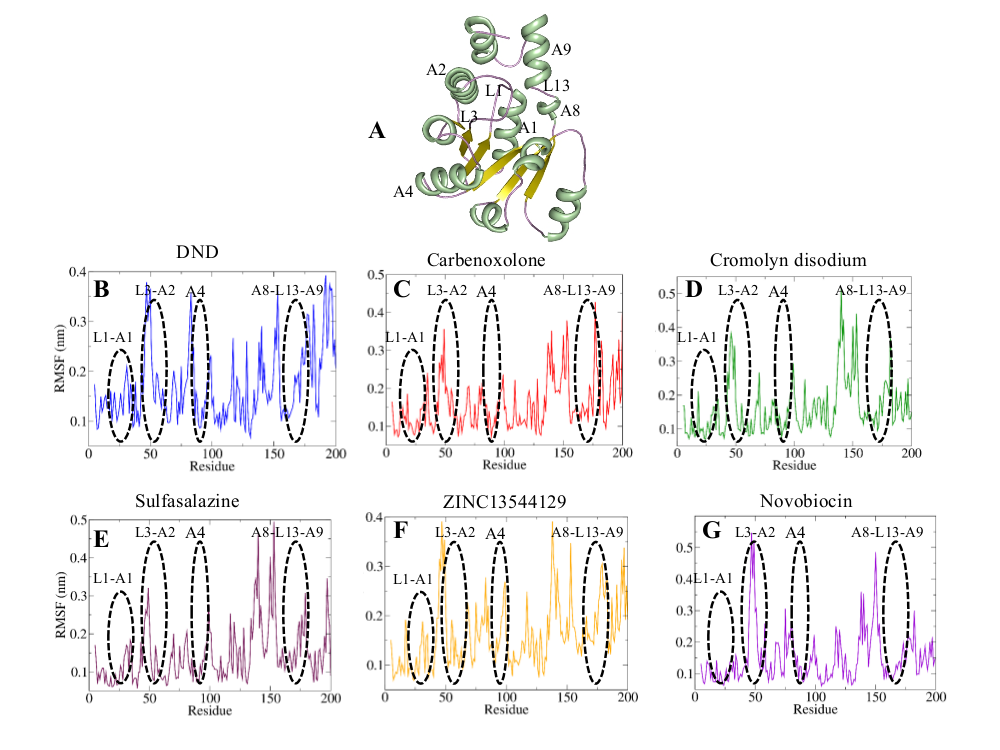

Supplement: S8 Fig — A) Structural depiction of active site residue fluctuation, B) DND, C) Carbenoxolone disodium salt, D) Cromolyn disodium salt, E) Sulfasalazine, F) ZINC13544129 and G) Novobiocin salt. (TIFF) [file pone.0259348.s008.tiff]
